# Serum neuron-specific enolase as predictor of outcome in comatose cardiac-arrest survivors: a prospective cohort study

**DOI:** 10.1186/1471-2261-11-48

**Published:** 2011-08-08

**Authors:** Cédric Daubin, Charlotte Quentin, Stéphane Allouche, Olivier Etard, Cathy Gaillard, Amélie Seguin, Xavier Valette, Jean-Jacques Parienti, Fabrice Prevost, Michel Ramakers, Nicolas Terzi, Pierre Charbonneau, Damien du Cheyron

**Affiliations:** 1 Department of Medical Intensive Care, CHU de Caen, Caen, F-14000, France; 2 Department of Medical Intensive Care, Mémorial France-Etats-Unis Hospital, Saint-Lô, France; 3 Department of Biochemistry, CHU de Caen, Caen, F-14000, France; 4 UPRES EA 3919, CHU de Caen, Caen University, Caen, F-14000, France; 5 Laboratory of Neurological Functional Exploratory, CHU de Caen, Caen, F-14000, France; 6 Department of Biostatistics and Clinical Research, CHU de Caen, Caen, F-14000, France; 7 INSERM, UMR-S 707, Paris, F-75012, France; 8 INSERM, ERI27, Caen, F-14000 France; Univ Caen, Caen, F-14000 France; CHRU Caen, Department of Medical Intensive Care, Caen, F-14000, France; 9 E.A. 4497, Université de Versailles-Saint Quentin en Yvelines, 92380 Garches, France; 10 UPRES, EA 2128, Caen, F-14000, France

## Abstract

**Background:**

The prediction of neurological outcome in comatose patients after cardiac arrest has major ethical and socioeconomic implications. The purpose of this study was to assess the capability of serum neuron-specific enolase (NSE), a biomarker of hypoxic brain damage, to predict death or vegetative state in comatose cardiac-arrest survivors.

**Methods:**

We conducted a prospective observational cohort study in one university hospital and one general hospital Intensive Care Unit (ICU). All consecutive patients who suffered cardiac arrest and were subsequently admitted from June 2007 to February 2009 were considered for inclusion in the study. Patients who died or awoke within the first 48 hours of admission were excluded from the analysis. Patients were followed for 3 months or until death after cardiopulmonary resuscitation. The Cerebral Performance Categories scale (CPC) was used as the outcome measure; a CPC of 4-5 was regarded as a poor outcome, and a CPC of 1-3 a good outcome. Measurement of serum NSE was performed at 24 h and at 72 h after the time of cardiac arrest using an enzyme immunoassay. Clinicians were blinded to NSE results.

**Results:**

Ninety-seven patients were included. All patients were actively supported during the first days following cardiac arrest. Sixty-five patients (67%) underwent cooling after resuscitation. At 3 months 72 (74%) patients had a poor outcome (CPC 4-5) and 25 (26%) a good outcome (CPC 1-3). The median and Interquartile Range [IQR] levels of NSE at 24 h and at 72 h were significantly higher in patients with poor outcomes: NSE at 24 h: 59.4 ng/mL [37-106] versus 28.8 ng/mL [18-41] (*p *< 0.0001); and NSE at 72 h: 129.5 ng/mL [40-247] versus 15.7 ng/mL [12-19] (*p *< 0.0001). The Receiver Operator Characteristics (ROC) curve for poor outcome for the highest observed NSE value for each patient determined a cut-off value for NSE of 97 ng/mL to predict a poor neurological outcome with a specificity of 100% [95% CI = 87-100] and a sensitivity of 49% [95% CI = 37-60]. However, an approach based on a combination of SSEPs, NSE and clinical-EEG tests allowed to increase the number of patients (63/72 (88%)) identified as having a poor outcome and for whom intensive treatment could be regarded as futile.

**Conclusion:**

NSE levels measured early in the course of patient care for those who remained comatose after cardiac arrest were significantly higher in patients with outcomes of death or vegetative state. In addition, we provide a cut-off value for NSE (> 97 ng/mL) with 100% positive predictive value of poor outcome. Nevertheless, for decisions concerning the continuation of treatment in this setting, we emphasize that an approach based on a combination of SSEPs, NSE and clinical EEG would be more accurate for identifying patients with a poor neurological outcome.

## Background

Despite improvement in resuscitation, the neurological outcome of comatose patients after cardiac arrest remains extremely poor [1]. Therefore, post-resuscitation anoxic encephalopathy represents a common problem with ethical, social, and legal consequences. In clinical practice, intensive care physicians are confronted with the ethical question of whether to continue treatment. In this context, providing predictors of poor outcome (death or permanent vegetative state) with a specificity of 100% could be useful for early identification of irrecoverable patients for whom intensive treatment could be regarded as futile and palliative care only could be given.

Currently, several clinical parameters and electro-encephalographic (EEG) patterns are recognised as being strongly associated with a poor outcome in unsedated comatose survivors of cardiac arrest; these include absence of pupillary or corneal reflexes, absence of extensor motor response to pain 3 days after cardiac arrest, myoclonus or epilepticus status within the first day after resuscitation, and a burst-suppression or isoelectric EEG pattern [1,2]. However, these clinical features and EEG readings could be severely affected by metabolic changes, therapeutic hypothermia or sedative drugs, limiting their clinical relevance for supporting a decision to withdraw active treatment. In contrast, bilateral absence of early cortical response to Somatosensory-Evoked Potentials (SSEPs) recorded on day 1 or later after cardiac arrest accurately predicts a poor outcome with 100% specificity, regardless of exam conditions [2-7]. However, this electrophysiological procedure is not routinely performed in all ICUs [8,9].

In this context, the serum Neuron-Specific Enolase (NSE), a biomarker of hypoxic brain damage which can be measured easily and reproducibly with minor invasiveness in patients, has recently been assessed as a prognostic predictor after cardiac arrest in several studies [2,9-23]. However, the cut-off points for predicting a poor outcome with no false positives vary greatly (9 to 91 ng/mL). Differences in definitions of poor outcome, the duration of follow up, the timing of blood sampling and assay procedures could explain these differences.

Therefore, we conducted a prospective cohort study to assess the capability of NSE, measured at fixed times, to predict a poor outcome (death or permanent vegetative state) with certainty in a predefined post cardiac-arrest comatose population.

## Methods

### Patients

We conducted a prospective cohort study of all consecutive non trauma patients who suffered out-of- or in-hospital cardiac arrest and were subsequently admitted to the adult intensive care unit in the Caen University Hospital and the Saint Lô General Hospital from June 2007 to February 2009. Patients who died or awoke within the first 48 hours of admission were excluded from this analysis. Therefore, only patients who remained in coma at 48 hours after cardiac arrest were included in the analysis. All patients were followed for 3 months after cardiac arrest or until death.

This study was submitted to the local ethics committee. The ethical board decided that approval was not necessary given the observational nature of this prospective study. Thus, in accordance with French legislation at the time of the study, no informed consent was obtained from the patients.

### Assessment of outcome

Neurological status at 3 months was assessed by telephone interview for patients discharged alive from the intensive care, using the 5-grade Glasgow-Pittsburgh Cerebral Performance Category (GP-CPC) scale [24]. *CPC 1*: conscious, alert, and oriented with normal cognitive functions, *CPC 2*: conscious and alert with moderate cerebral disability, *CPC 3*: conscious with severe disability, *CPC 4*: comatose or in persistent vegetative state, *CPC 5*: certified brain death or dead by traditional criteria. A CPC score of 1-3 was considered a good outcome and a CPC of 4-5 a poor outcome.

### Measurement of serum NSE

Blood samples were collected at 24 h and 72 h after the time of cardiac arrest. All samples with visible hemolysis were discarded from analysis to avoid any falsely elevated values for serum NSE. Blood was centrifuged at 3 000 rpm for 10 min. The isolated serum was immediately frozen at -80°C and stored until time of assay. The serum NSE level was measured using a solid-phase immunoassay with double monoclonal antibodies directed against NSE (Roche Diagnostics GmbH, Mannheim, Germany) on an Elecsys instrument. The limit of detection was 0.05 ng/mL and the institutional normal value was < 16.3 ng/mL. When the NSE level reached 50 ng/mL, the serum was diluted to avoid a hook effect. Clinicians were blinded to NSE results during the entire patient stay. For each patient, the highest measurement of NSE was tested for outcome prediction. Because the 24 h value is more variable than that at 72 h, we also considered the 72 h NSE value as the highest observed NSE in the sensitivity analysis.

### Data collection

Clinical variables collected at baseline were: age, sex, underlying diseases, cause of the arrest (cardiac, respiratory, other or unknown), time between arrest and cardiopulmonary resuscitation, initial cardiac rhythm (ventricular fibrillation or tachycardia, asystole, pulseless rhythm), duration of cardiopulmonary resuscitation, number of external electric shocks, cumulative epinephrine dose, and scoring of disease severity within the first day in ICU as assessed during admission by the Simplified Acute Physiology Score type II (SAPS II) [25], and number of organ failures according to Knaus criteria [26].

Following our standard of treatment [27], a neurological assessment was daily performed after cardiac arrest using measures of clinical and electrophysiological evaluation. For patients who underwent cooling after resuscitation, clinical and neurophysiological tests, including EEG and SSEPs recording, were performed after warming. For this reason, the findings of the first neurological assessment reported in results section were recorded within 24 h-36 h after resuscitation.

Clinical parameters included pupillary light reflex (present/absent), motor response to painful stimulation (extensor or absent response/other response), corneal reflex (present/absent), tonic-clonic seizures (present/absent) and myoclonus (present/absent).

Electrophysiological assessment included EEG and Somatosensory-Evoked Potential (SSEP) recordings, routinely performed in our centre. All EEGs and SSEPs were read by an expert neurophysiologist (O.E.). Two EEG were performed: the first within 24 h-36 h after resuscitation and the second at 72 h. EEGs were recorded on a system with at least 10 channels and needle electrodes and used a 10-20 international system (Fp1, Fp2, C3, C4, T5, T6, O1, and O2). The EEG patterns were classified according to the classification system of Synek et al. [28,29]. EEG results were categorised as either malignant (isoelectric, burst-suppression pattern with interburst interval of at least 1 s and generalised continous epileptiform discharges) or non malignant (other patterns, including alpha and theta coma). SSEPs were performed as soon as possible after the first 24 hours after resuscitation. However, if SSEPs recording was due on a weekend day, the recording was postponed to Monday. SSEPs were recorded on a Medtronic keypoint system using 6 channels: erb'point; C6sp; C'3 or C'4, contralateral to the stimulated hand and Fpz (ipsilateral ear was used as a reference). The 2 remaining channels served as channel controls: C'3 - C'4 (or C'3-C'4) on which the N20 amplitude was measured, and Fpz-C'3 (or Fpz-C'4) in order to check for a long latency component using a larger time window. Absence of early cortical responses to somatosensory-evoked potentials (N20) were declared only if the 3 following conditions were present: (i) correct peripheral (N10) and medullary (N13) component, (ii) no deflection higher than 0.5μV on C3-C'4 (or C'3-C'4), (iii) no late component on Fpz-C'3 (or Fpz-C'4).

### Treatment and treatment restriction

All patients were actively supported during the first days following cardiac arrest or until SSEP assessment. They received standard intensive care management and monitoring. In addition, in our practice, therapeutic hypothermia (target temperature 33°C) was recommended for all cardiac causes of arrest, regardless of initial rhythm, and left to the assessment of the attending physician for the other causes. The patients who underwent cooling received propofol or midazolam and sufentanyl for sedation and atracurium (0.5 mg/kg per hour) to prevent shivering. These drugs were stopped after passive rewarning to a central temperature of 36°C. Hypothermia was induced using an endovascular cooling catheter (IcyTM, Alsius, Irvine, CA, USA) inserted into the inferior vena cava via the femoral vein and connected to a cooling device (Coolgard 3000TM, Alsius, Irvine, CA, USA), and was maintained for 24 h. In patients with a bilateral lack of cortical response (N20) to SSEPs, further treatment was considered futile and active care was withdrawn. In addition, in accordance with our previous report [27] and recent literature [1,2,30] a decision to withdraw treatment was debated by the medical staff after 5 days in patients with 3 or more pejorative criteria at 72 h; these included: absence of pupillary light reflex or corneal reflex, extensor or absence of motor response to painful stimulation, persistent myoclonus, and a malignant EEG pattern

### Statistical analysis

Categorical variables were reported as counts and percentages. Quantitative data which did not follow a Gaussian curve (NSE levels) were described as median and Interquartile Range [IQR, first quartile - third quartile]. Other qualitative data were reported as mean ± standard deviation (SD). Means were compared between two groups using Student's *t*-test, and median levels of NSE were compared between two groups using the Mann-Whitney U test. Percentages were compared using a chi-square test or Fisher exact test for small samples. The discriminative power of the highest measurement of NSE in predicting poor outcome at 3 months (CPC 4-5) was evaluated by Receiver Operating Characteristic (ROC) analysis. Thresholds for NSE are given for 100% specificity and the highest sensitivity. Area Under the Curve (AUC) is given with 95% confidence intervals. Finally, the percentage of abnormal test results, false positive rates and positive likelihood ratios with their 95% confidence interval were calculated. The positive likelihood ratio calculation was made possible by adding 0.5 in the cell when there was no patient with a poor test result and a good outcome [31]. We used EPI-INFO version 6.04 dfr (EPI-INFO, CDC, Atlanta, GA) for data collection, and EPI-INFO and SAS version 9.1 (SAS Institute Inc, Cary, NC) for data analysis

## Results

### Baseline characteristics and neurological outcome

**Figure 1:**
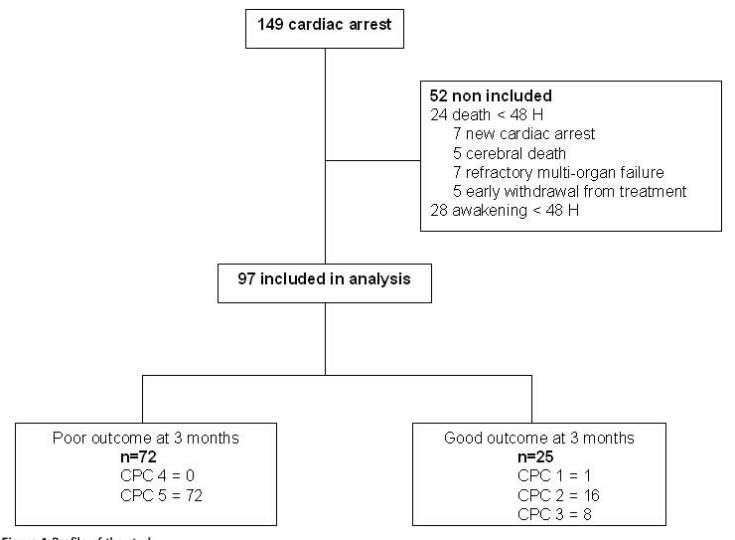
**Profile of the study**.

Of the 149 consecutive patients resuscitated after a cardiac arrest and admitted to the intensive care unit during the study period, 97 fulfilled inclusion criteria for analysis. At 3 months 72 (74%) patients had a poor outcome (CPC 4-5) and 25 (26%) had a good outcome (CPC 1-2, n = 17 and CPC 3, n = 8) as shown in Figure 1. All but one death occurred in the ICU, and in 93% of the cases they were associated with a decision to withdraw active treatment. In patients (n = 67) for whom a decision to withdraw active treatment was taken, the survival median time was 5 days [IQR, 4-8]. As expected in these patients, the survival median time was lower in patients (n = 45) with unfavorable SSEP results (4 days [IQR, 3-5]) than in those (n = 22) with favorable SSEP results (13 days [IQR, 7-20]), p = 0.02.

Patient baseline characteristics are presented in Table 1. Patients with a poor outcome were older, with more frequent histories of coronary disease or diabetes mellitus and had higher disease severity scores at admission. However, regarding resuscitation time, there was no difference between patients with a good or poor outcome, except for primary cause of cardiac arrest. Sixty-five patients (67%) underwent cooling after resuscitation: 45 in group CPC 4-5 and 20 in group CPC 1-3, *p *= 0.11.

**Table 1:** Baseline characteristics

	All patients n = 97	Poor outcome n = 72	Good outcome n = 25	p
**Age (years), mean ± SD**	57 ± 16	60 ± 15	50 ± 17	0.01
**Male, n (%)**	75 (77)	56 (78)	19 (76)	0.85
**Medical history n (%)**				
Neurologic diseases	11 (11)	10 (14)	1 (4)	0.18
Cardiovascular diseases				
Ischemic	20 (21)	19 (26)	1 (4)	0.017
Hypertensive	34 (35)	29 (40)	5 (20)	0.067
Congestive	12 (12)	10 (14)	2 (8)	0.44
Arrhythmic	4 (5)	3 (4)	2 (8)	0.82
Metabolic diseases				
Diabetes mellitus	17 (18)	17 (24)	0	0.007
Respiratory diseases				
COPD	7 (7)	6 (8)	1 (4)	0.47
Liver diseases				
Cirrhosis	4 (4)	4 (6)	0	0.53
**Resuscitation variables**				
Witnessed CA n (%)	77 (79)	57 (79)	20 (80)	0.93
In-hospital CA n (%)	30 (31)	26 (36)	4 (16)	0.06
Primary cause of CA n (%)				
Cardiac	55 (57)	38 (53)	17 (68)	0.19
Respiratory	17 (18)	16 (22)	1 (4)	0.04
Other or unknown	22 (23)	17 (24)	5 (20)	0.7
Time from CA to CPR (minutes)	5.6 ± 6.9	6.3 ± 7.2	3.4 ± 5.5	0.07
<3 n (%)	46 (51)	31 (46)	15 (65)	
3 -5 n (%)	9 (10)	6 (9)	3 (13)	
>5 n (%)	36 (40)	31 (46)	5 (22)	
Duration of CPR (minutes)	24.9 ± 24.2	25.7 ± 26.6	22.5 ± 15.7	0.39
<5 n (%)	9 (9)	5 (7)	4 (16)	
5 -15 n (%)	18 (19)	13 (18)	5 (20)	
>15 n (%)	69 (72)	53 (75)	16 (64)	
Primary rhythm n (%)				0.09
Asystole	55 (57)	46 (64)	9 (36)	
VF/VT	35 (36)	21 (29)	14 (56)	
Pulseless electrical activity	5 (5)	4 (6)	1 (4)	
Unknown	2 (2)	1 (1,4)	1 (4)	
Number of defibrillations, mean ± SD	2.1 ± 3.1	1.9 ± 3.3	2.8 ± 2.4	0.17
Epinephrine mg mean, ± SD	5.1 ± 5.2	5 ± 4.8	5.6 ± 6.2	0.62
**ICU admission**				
SAPS II mean, ± SD	67 ± 15	69 ± 15	61 ± 11	0.006
Shock n(%)	59 (61)	43 (60)	16 (64)	0.7
Renal replacement therapy n(%)	15 (16)	11(15)	4 (16)	1
MOF n(%)	18 (19)	16 (22)	2 (8)	0.14
Therapeutic hypothermia n(%)	65 (67)	45 (63)	20 (80%)	0.11

CA, cardiac arrest; COPD, chronic obstructive pulmonary disease; CPR, cardiopulmonary resuscitation; FV ventricular fibrillation; MOF, multi organ failure according to Kraus criteria [27]; SAPS II, Simplified Acute Physiology Score type II; VT, ventricular tachycardia.

### Serum NSE levels and prediction of poor outcome

**Figure 2:**
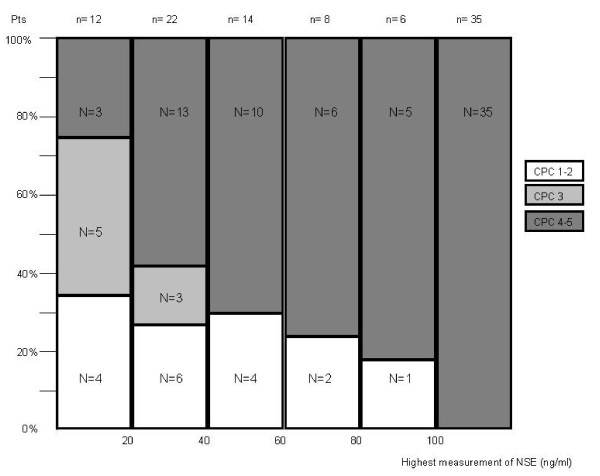
**Frequency of different neurological outcome categories in relation to the individual highest measurement of NSE**.

The median levels of NSE at 24 h (n = 86) and at 72 h (n = 61) and the highest measurement of NSE for each patient were significantly higher in patients with poor outcomes: median 24 h NSE level: 59.4 ng/mL [IQR, 37-106] versus 28.8 ng/mL [IQR, 18-41] (*p *< 0.0001); median 72 h NSE level: 129.5 ng/mL [IQR, 40-247] versus 15.7 ng/mL [IQR, 12-19] (*p *< 0.0001); and highest measurement of NSE: 87.6 ng/mL [IQR, 44-178] versus 28.8 ng/mL [IQR, 19-41] (*p *< 0.0001). The frequency of different neurological outcome categories in relation to the highest individual measurement of NSE concentration of our patients is presented Figure 2.

In our sample group, the highest measurements of NSE for each patient were not different in patients treated with or without induced hypothermia: 67.4 ng/mL [IQR, 37.2-143.6] versus 55.3 ng/mL [IQR, 25.1-159.4] (*p *= 0.7). In addition, no significant difference for the highest measurement of NSE was observed in patients with a good outcome (CPC 1-3) treated with (n = 20) or without (n = 5) hypothermia; highest measurement of NSE: 29.6 ng/mL [IQR, 18.7-46.3; Range, 9.7-91.7 ] versus 19.6 ng/mL [IQR, 8.5-25.5; Range, 8-37.4], p = 0.02, respectively. A similar result was observed in patients with a poor outcome (CPC 4-5): highest measurement of NSE in patients (n = 45) treated with hypothermia 105.7 ng/mL [IQR, 48.5-179.7; Range, 16.7-952.3] versus 82.8 ng/mL [IQR, 32.5-172.2; Range, 19.5-1071] in patients (n = 27) without therapeutic hypothermia p = 0.02.

**Figure 3:**
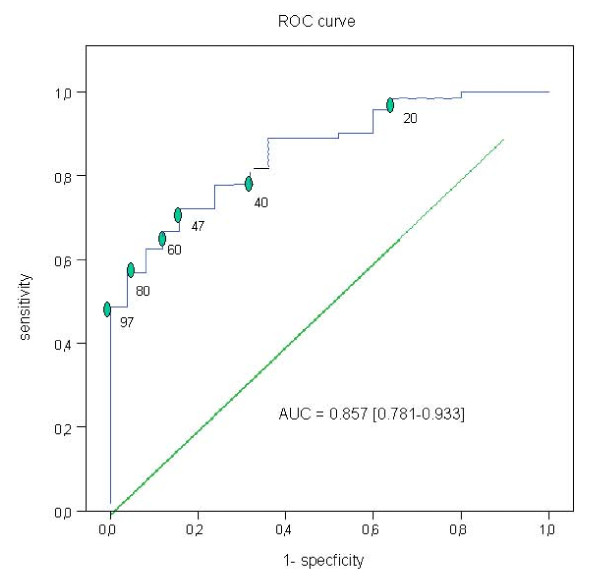
**Receiver operating characteristic curves for different peak serum NSE cut-off values (ng/mL) to predict poor neurological outcome**.

The ROC curve for poor outcome for the highest measurement of NSE for each patient is presented in Figure 3. The NSE level with the highest specificity and sensitivity was 47 ng/mL (Sp = 84% [95% CI = 70-98], Se = 72% [95% CI = 62-83]) with a positive predictive value of 93% [95% CI = 86-100]. An NSE level ≥ 97 ng/mL predicted a poor outcome with a positive predictive value of 100% [95% CI = 87-100] and a sensitivity of 49% [95% CI = 37-60]. Interestingly, 32/35 (91%) and 29/35 (83%) of patients with a NSE measurement > 97 ng/mL had unfavorable SSEP results and a malignant EEG pattern at 72 h, respectively, and the 3 and 6 remaining patients had 3 or more unfavorable clinical and electrophysiological criteria, respectively (see below).

The sensitivity analysis provided similar results (see additional file 1: " Receiver operating characteristic curves for 72 h NSE value (ng/mL) to predict poor neurological outcome")

Contribution of each clinical, electrophysiological and biological test to prediction of poor outcome

The absence of cortical response to SSEPs was recorded in 45 patients. An unfavorable SSEP result was associated with 2.9 ± 1 predefined unfavorable clinical-EEG criteria. In accordance with our treatment restriction policy, all died.

The predictive values of clinical-EEG and biological tests for a poor outcome are presented in Table 2. An NSE level ≥ 97 ng/mL (*n *= 35), myoclonus at 24 h (*n *= 26), and absence of pupillary light reflex (*n *= 18) or corneal reflex (*n *= 31), tonic-clonic seizures (*n *= 6) and malignant EEG pattern at 72 h (*n *= 32) were predictive for a poor outcome with no false positives. Electrophysiological and biological tests had a higher percentage of abnormal test results than clinical tests.

**Table 2:** Prediction of poor outcome with clinical, electrophysiological and biological variables

	Patients tested, n	Abnormal test result, % (95% CI)	False positive rate*, % (95% IC)	Positive likelihood ratio (95% IC)
Motor response ≤ 2				
- 24 h-36 h	97	87% [80% - 93%]	56% [45%- 67%	1.7 [1.2 - 2.5]
- 72 h	87	70% [61% - 80%]	16% [7% - 25%]	5.8 [2.3 -1 4.1]
				
No corneal reflexes				
- 24 h-36 h	95	40% [30% - 50%]	12% [2% - 22%]	4.2 [1.4 - 12.4]
- 72 h	86	36% [26% - 46%]	0% [0%-14%]	26 [1.7 - 415]
No pupillary reflexes				
- 24 h-36 h	97	26% [17% - 35%]	4% [4% - 12%]	8.3 [1.2 - 58]
- 72 h	87	21% [12% - 29%]	0% [0% - 14%]	15 [1 - 244]
Myoclonus				
- 24 h-36 h	97	27% [18% - 36%]	0% [0% -14%]	19 [1.2 - 299]
- 72 h	87	23% [14% - 32%]	8% [4% - 20%]	3.6 [1-14]
Epilepsy				
- 24 h-36 h	97	7% [7% - 12%]	0% [0% -14%]	5.3 [0.3-90]
- 72 h	87	7% [2% - 12%]	0% [0% -14%]	5.3 [0.31-91]
- Malignant EEG **				
- 24-36 h	90 ^¶^	44% [34% - 55%]	4% [2% - 10%]	14 [2.1-97]
- 72 h	73^¶¶^	44% [33% - 55%]	0% [0% -17%]	25 [1.6-394]
Highest measurement of NSE ≥ 47 ng/mL				
	97	58% [48% - 68%]	7% [0,4% - 14%]	4.5 [1.8 -11]
Highest measurement of NSE ≥ 97 ng/mL				
	97	36% [27% - 46%]	0% [0% -13%]	25 [1.6-397]

* Patients with abnormal test results and good outcome/all patients with abnormal test results (1-positive predictive value)

** included isoelectric and burst-suppression pattern with interburst interval of at least 1s and generalised continuous epileptiform discharges

^¶ ^Forty-four patients were still affected by sedative medication

^¶¶ ^twenty-one patients were still affected by sedative medication

**Figure 4:**
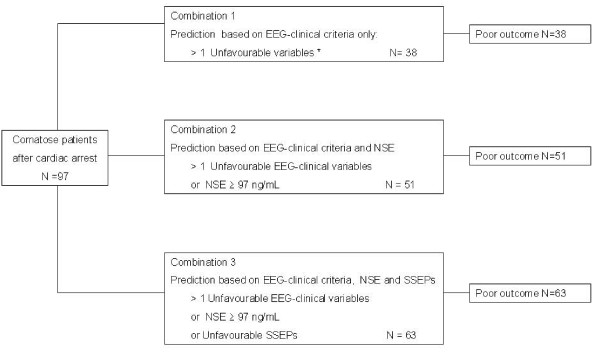
**Predictors of poor outcome according to different clinical-EEG, NSE and SSEPs combinations**. * included: myoclonus at 24 h, and absence of pupillary light reflex or corneal reflex, tonic-clonic seizures and malignant EEG pattern at 72 h.

An approach based on a combination of SSEPs, NSE and clinical-EEG tests increased the number of patients (63/72 (88%)) identified as having a poor outcome, as shown in Figure 4.

No tests predicted a good outcome. For example, only 51% (24/47) of our patients with a favorable SSEP result made a good recovery

## Discussion

Considering the poor neurological prognosis of comatose patients after cardiac arrest, physicians are rapidly confronted with the ethical question of whether to continue intensive treatment. To our knowledge, except for unfavorable SSEP results, predictors of poor outcome with a 100% specificity and a high sensitivity are lacking [1,2,8]. Recently, several studies [2,9-23] have investigated the usefulness of increased serum NSE as a marker of poor outcome. In this prospective study, we provide a cut-off value for NSE (> 97 ng/mL), measured at a fixed time after cardiac arrest, with 100% predictive value for a poor neurological outcome (death or vegetative state). However, a strategy based on a combination of SSEPs, NSE and clinical-EEG tests increase the number of patients identified as having a poor outcome. These results may have important implications in determining the level of care to be provided three days after cardiac arrest, involving SSEPs or NSE access, and clinical EEG evaluation.

In our study, 74% of the patients who remained comatose after cardiac arrest never regained consciousness. This result is consistent with previous studies [2,32-36] and with our recent publication [27] reporting an early clinical and electrophysiological score with 100% predictive value for poor outcome in this setting. The proportion of patients (49%) with no early cortical responses to SSEPs, a result recognized to be the most accurate predictor of a poor outcome in survivors after a cardiac arrest [2,3,8,34,35,37-39], is also consistent with previous reports [2,27,34-36]. We also report that an unfavorable SSEP result was associated with a mean of 2.9 ± 1 other pejorative clinical-EEG criteria. In addition, our results confirm that the presence of early cortical responses to SSEPs is a poor predictor of a good outcome, as previously reported [2,27,34,40]; indeed, only 51% (24/47) of our patients with a favorable SSEP result made a good recovery. Interestingly, we report that the absence of motor response to painful stimuli has a higher false positive poor outcome prediction compared to the American Academy of Neurology (AAN) meta-analysis [1] and that a malignant EEG is strongly associated with outcome. These findings are consistent with a recent report [41] assessing the prognostic value of clinical and electrophysiological variables in comatose survivors of cardiac arrest treated with therapeutic hypothermia and suggest using caution in the application of AAN guidelines.

NSE levels were significantly higher in comatose cardiac arrest survivors with poor outcome, regardless of the time of measurement. In addition, we identified a cut-off value for NSE (> 97 ng/mL), predicting a poor outcome (death or vegetative state) with no false positives. Interestingly, 32/35 (91%) and 29/35 (83%) patients with an NSE measurement > 97 ng/mL had an unfavorable SSEP result and a malignant EEG pattern at 72 h, respectively. These findings argue that NSE measurement may be used, in combination with clinical tests, as a potential substitute for SSEPs and EEG in settings without access to electrophysiological assessment, for early identification of a subgroup of irrecoverable patients for whom continued intensive care could be considered futile.

However, the cut-off values for NSE with 100% predictive value of poor outcome determined in our study is rather high in comparison with others (range 9 to 91 ng/mL) previously reported (Table 3). Differences in selected patients (outside- or inside-hospital resuscitation, primary cause of cardiac arrest, initial rhythm, time of inclusion after cardiac arrest), definitions of poor outcome (death, Glasgow Coma Scale < 8, absence of regaining of consciousness (CPC 4-5) or without return to normal social activity (CPC 3-5)), duration of follow up (hospital discharge, 1-month, 3-month or 6-month), time of blood sampling and assay procedures could explain these differences. Nevertheless, we consider that a poor outcome defined by CPC 4-5 is more accurate in a study assessing a cut-off value of a biomarker on which treatment withdrawal could be decided. In this context, except for references [2] and [10], cut-off values for NSE are highest (range 39 to 91 ng/mL) in studies [15-19] with vegetative state or death as endpoints, in accordance with our results.

**Table 3:** Comparison of NSE levels to predict poor outcome after cardiac arrest and referenced study profiles

Référence	In-hospital CPR (%)	Time of inclusion after CPR, n	Hypothermic therapy, n	Follow-up	Poor outcome definition, and number n	Method used for NSE measurement	NSE sampling time	Cut-off value (ng/mL)	Se	Sp
Fogel et al. [10] 1997	Not specified	ICU admission n = 43	No	3 months	Remained comatose n = 25	Radioimmunoassay, Pharmacia LKB	Day 0 Day 1 Day 2 Day 3	**33** **33** **33** **33**	25 60 63 65	100 100 100 100

Martens et al. [21] 1998	Not specified	> 24 h n = 64	No	6 months	Remained comatose n = 35	Radioimmunoassay, Profilogen	24 h	**20**	51	89

Schoerkhber et al. [11] 1999	Yes (not specified)	> 6 h n = 56	No	6 months	CPC 3-5 n = 28	Radioimmunoassay, Profilogen	12 h 24 h 48 h 72 h Peak NSE level 12-72 h	**38** **40** **25** **17** **27**	18 8 48 70 29	100 100 100 100 100

Rosen et al. [12] 2001	No	> 24 h n = 66	No	1 year	CPC 3-5 n = 42	Imunoluminometric assay, Byk Sangtec Diagnostica	Day1 Day2 Day3	**25** **25** **25**	NM NM NM	100 100 100

**Zingler et al**. [15]**2003**	Yes (not specified)	ICU admission n = 27	No	3 months	**CPC 4-5** **n = 17**	Immunoluminometric assay, Byk Sangtec Diagnostica	Day1 Day2 Day3 Day7	**48** **43** **91** **39**	53 91 75 57	100 100 100 100

Tiainem et al. [14] 2003	No*	ICU admission n = 70	Yes n = 36	6 months	CPC 3-5 n = 29	time-resolved immunofluorometric assay (DELFIA, Wallac)	24 h HT/no HT 36 h HT/no HT 48 h HT/no HT	**31/13** **26/13** **25/9**	22/59 30/63 25/76	96/100 96/100 96/100

Meynaar et al. [13] 2003	Yes (23%)	ICU admission n = 110	No	Hospital discharge	Remained comatose n = 81	time-resolved immunofluorometric assay (DELFIA, Wallac)	Peak NSE level 24-48 h¶	**25**	59	100

**Pfeifer et al**. [16]**2005**	Yes (44%)	> 48 h n = 97	Not specified	Day 28	**CPC 4-5** **n = 70**	Imunoluminometric assays, Byk Sangtec Diagnostica	Day 3	**65**	50	96

**Rech at al**. [17]**2006**	Yes (100%)	>12 h n = 45	No	6 months	**CPC 4-5** **n = 34**	Electrochemiluminescence immunoassay, Roche **Mannheim**	Between 12-36 h	**60**	35	100

**Zandbergen et al** [2]**2006**	Not specified	> 24 h n = 407	Yes (not specified)	1 months	CPC 4-5 n = 356	Immunoluminometric assay, Byk Sangtec Diagnostica	24 h 48 h 72 h	**>33** **>33** **>33**	42 52 46	100 100 100

Auer et al. [18] 2006	Yes (not specified)	n = 17	Not specified	Hospital discharge	Death n = 9	Electrochemiluminescence immunoassay, Roche **Mannheim**	48 h	**30**	79	100

Grubb et al. [23] 2007	No	ICU admission n = 143	Not specified	Hospital discharge	Death	Enzyme immunoassay, Roche Diagnostics	12 h 24-48 h 72-96 h	**NM** 71 **NM**	14	100

**Reisinger et al**. [19]**2007**	Yes (44%)	ICU admission n = 177	Yes n = 20	6 months	**CPC 4-5** **n = 59**	Electrochemiluminescence immunoassay, Roche **Mannheim**	Peak NSE level Day 0-4	**80**	63	100

Oksanen et al. [20] 2009	No*	ICU admission n = 90	Yes n = 90	6 months	CPC 3-5 n = 40	Electrochemiluminescence immunoassay, Roche **Mannheim**	24 h 48 h	**41** **33**	20 43	100 100

Rundgren et al. [9] 2009	Yes (17%)	ICU admission n = 102 (for NSE cohort)	Yes n = 102	6 months	CPC 3-5 n = 46	Imunoluminometric assay, DiaSorin	2 h 24 h 48 h 72 h	**31** **38** **28** **27**	6 11 67 50	100 100 100 100

Shinozaki et al. [22] 2009	Yes (27%)	ICU admission n = 80	Yes n = 45	6 months	CPC 3-5 n = 67	Immunoradiometric assay, Profilogen, DiaSorin	Admission 6 h 24 h	**46** **66** **40**	14 19 72	100 100 100

Steffen et al [42] 2010	Yes (21%)	ICU admission n = 240	Yes n = 133	ICU discharge	**CPC 3-5** n = 147	Electrochemiluminescence immunoassay, Roche **Mannheim**	72 h HT/no HT	**79/27**	50 80	100 100

**Present study**	**Yes (31%)**	**> 48 h** **n = 97**	**Yes** **n = 65**	**3 months**	**CPC 4-5** **n = 72**	Electrochemiluminescence immunoassay, Roche **Mannheim**	**Peak NSE level 24-72 h**	**97**	**49**	**100**

HT; hypothermic therapy, NM; not mentioned

* Only ventricular fibrillation as initial rhythm was eligible for the study

^¶ ^Results mentioned only for patients who remained comatose 48 hours after CPR (n = 67)

Whether hypothermia, performed in most of our patients, may affect NSE measurement is unclear. Recently, studies [9,20] testing the predictive values for NSE to predict poor outcome (CPC 3-5) in comatose patients after cardiac arrest treated with therapeutic hypothermia, reported cut-off values for NSE, at 48 h, of 28 ng/mL and 33 ng/mL, respectively; a result no different from that reported by Zandbergen et al. in patients without therapeutic hypothermia [2]. In contrast, hypothermic therapy for prevention of hypoxic damage after cardiac arrest has been reported to decrease NSE levels in comparison with levels with normothermic therapy [14,42]. However, in these studies [14,42], the cut-off values for NSE predicting a poor outcome (CPC 3-5) were significantly higher in patients treated with induced hypothermia than in patients without therapeutic hypothermia. In this setting, NSE levels were higher (but not significantly) in patients treated with induced hypothermia.

Therefore, considering decisions to continue treatment in comatose patients after cardiac arrest, the different cut-off values for NSE reported in the literature should be interpreted with caution as many patient, treatment, and assay-procedure related factors may influence NSE measurement. For example, if we had used the NSE threshold > 33 ng/mL, defined by Zandbergen et al. [2] to withdraw treatment in patients remaining comatose 24 h after cardiac arrest, 10/25 (40%) of our patients with a good outcome could have had false predictions of a poor outcome (death or vegetative state).

We also report that an approach based on a combination of electrophysiological, biological and clinical tests allowed to increase the number of patients identified as having a poor outcome (Figure 3), in accordance with other studies [2,8,13,15,16,19,27]. Therefore, based on all available evidence [1-24,27,30,32-40] and our current results, we suggest the following strategy to help clinicians determine the level of care to be provided in patients remaining comatose three days after cardiac arrest: when the cortical response (N20) to SSEPs is bilaterally absent, further treatment should be considered futile and active care withdrawn. When the SSEP recording is equivocal, it must be repeated. When SSEPs are favourable or when SSEPs are not accessible, the presence of more than one pejorative EEG-biological-clinical criterion at day 3 should be considered sufficient to forego further treatment; these include: serum NSE > 97 ng/mL, malignant EEG pattern (burst-suppression with or without epileptiform discharge or isoelectric pattern), absence of pupillary light reflex or corneal reflex, and persistence of tonic-clonic seizures or myoclonus. Moreover, we would like to emphasize that no tests are available that can reliably predict recovery of consciousness or the quality of life in survivors. Nevertheless, a recent study reported that continuous amplitude-integrated electroencephalogram added valuable positive and negative prognostic information in hypothermia-treated cardiac arrest patients [43].

This study has some limitations. Firstly, the relative small sample size may limit the interpretation and relevance of the cut-off value for NSE. In addition, because of differences in NSE measurements obtained using assays from different manufacturers (Table 3), the cut-off values for NSE reported in this setting should be interpreted with caution. Secondly, hemolysis due to renal replacement therapy needed for 15 patients at ICU admission may affect the cut-off value for NSE. However, we believe the effect of this hemolysis was limited because the highest measurement of NSE was significantly lower in patients with renal replacement therapy (59 ng/mL [IQR, 35-112] versus 67 ng/mL [IQR, 34-159], *p *= 0.025). Thirdly, the applicability of our findings could be limited because of early withdrawal of treatment for patients with poor prognoses, which could result in a self-fulfilling prophesy of poor outcome. However, all of our patients were actively supported without restriction during the first days following cardiac arrest or until SSEPs were assessed, and only a lack of bilateral cortical response to SSEPs associated with pejorative clinical-EEG criteria in all cases in our study, or the presence of 3 or more predefined pejorative clinical-EEG criteria, recognised to predict a worse outcome [1,2,30], could lead to active care withdrawal. In addition, because our study focuses specifically on comatose patients after cardiac arrest for whom there is an ethical question of whether to continue treatment, we believe that this report adds useful information about clinical outcome and predictors of death or vegetative state in this setting.

## Conclusion

We show that NSE levels, measured early in the course of patient care for those who remained comatose 3 days after cardiac arrest, are significantly higher in patients with a poor outcome (death or vegetative state). In addition, we provide a cut-off value (> 97 ng/mL) for NSE, with 100% predictive value for a poor outcome. Nevertheless, considering decisions to continue treatment in this setting, we emphasize that cut-off values for NSE presented in the literature should be interpreted with caution and that an approach based on a combination of SSEPs, NSE and clinical-EEG tests would be the most accurate for early identification of a subgroup of irrecoverable patients for whom intensive treatment could be regarded as futile and palliative care only could be provided.

## Abbreviations

EEG: Electro-Encephalography; GPCPC: Glasgow-Pittsburgh Cerebral Performance Category; NSE: Neuron-Specific Enolase; SSEPs: Somatosensory Evoked Potentials; ROC: Receiver Operating Characteristics.

## Competing interests

The authors declare that they have no competing interests.

## Authors' contributions

CD and CQ initiated the study, and the design. SA performed NSE measurement. OE read all EEGs and SSEPs. JJP, CG and CD performed the statistical analysis and were involved in the interpretation of the results. CD and CQ wrote the manuscript, and JJP and PC helped to draft the manuscript. AS, XV, FP, MR, NT, OE, SA and DDC, contributed to the conception and design of the study and revision of the manuscript. All authors read and approved the final manuscript.

## Author information

This work was presented in part at the annual congress of the Société de Réanimation de Langue Française (SRLF) held in January 2010, Paris, France.

This study was funded by an academic unrestricted grant (Appel d'Offre Interne) from the Caen Côte de Nacre University hospital.

## Pre-publication history

The pre-publication history for this paper can be accessed here:

http://www.biomedcentral.com/1471-2261/11/48/prepub

## Supplementary Material

Additional file 1 **" Receiver operating characteristic curves for 72 h NSE values (ng/mL) to predict poor neurological outcome**. This file highlight that considering 72 h-NSE values (n = 61), a level ≥ 68 ng/mL predicted a poor outcome (CPC 4-5) with a positive predictive value of 100% [95% IC = 100% - 100%] and a sensitivity of 67% [95% IC = 54% - 81%]. Click here for file

## References

[B1] Wijdicks Neurology 2006 13these Wijdicks EF Hijdra A Young GB Bassetti CL Wiebe S Practice parameter: prediction of outcome in comatose survivors after cardiopulmonary resuscitation (an evidence-based review): report of the Quality Standards Subcommittee of the American Academy of Neurology Neurology 2006 67 203 210 10.1212/01.wnl.0000227183.21314.cd 16864809

[B2] Zandbergen EG Hijdra A Koelman JH Hart AA Vos PE Verbeek MM de Haan RJ PROPAC Study Group Prediction of poor outcome within the first 3 days of postanoxic coma Neurology 2006 66 62 68 16401847 10.1212/01.wnl.0000191308.22233.88

[B3] Tiainen M Kovala TT Takkunen OS Roine RO Somatosensory and brainstem auditory evoked potentials in cardiac arrest patients treated with hypothermia Crit Care Med 2005 33 1736 1740 10.1097/01.CCM.0000171536.63641.D9 16096450

[B4] Koht A Schatz W Schmidt G Schramm J Watanabe E Effects of etomidate, midazolam, and thiopental on median nerve somatosensory evoked potentials and the additive effects of fentanyl and nitrous oxide Anesth Analg 1988 67 435 441 3364762

[B5] Sloan TB Fugina ML Toleikis JR Effects of midazolam on median nerve somatosensory evoked potentials Br J Anaesth 1990 64 590 593 10.1093/bja/64.5.590 2354098

[B6] Stecker MM Cheung AT Pochettino A Kent GP Patterson T Weiss SJ Bavaria JE Deep hypothermic circulatory arrest: I. Effects of cooling on electroencephalogram and evoked potentials Ann Thorac Surg 2001 71 14 21 10.1016/S0003-4975(00)01592-7 11216734

[B7] Kottenberg-Assenmacher E Armbruster W Bornfeld N Peters J Hypothermia does not alter somatosensory evoked potential amplitude and global cerebral oxygen extraction during marked sodium nitroprusside-induced arterial hypotension Anesthesiology 2003 98 1112 1118 10.1097/00000542-200305000-00013 12717132

[B8] Zandbergen EG de Haan RJ Stoutenbeek CP Koelman JH Hijdra A Systematic review of early prediction of poor outcome in anoxic-ischaemic coma Lancet 1998 352 1808 1812 10.1016/S0140-6736(98)04076-8 9851380

[B9] Rundgren M Karlsson T Nielsen N Cronberg T Johnsson P Friberg H Neuron specific enolase and S-100B as predictors of outcome after cardiac arrest and induced hypothermia Resuscitation 2009 80 784 789 10.1016/j.resuscitation.2009.03.025 19467754

[B10] Fogel W Krieger D Veith M Adams HP Hund E Storch-Hagenlocher B Buggle F Mathias D Hacke W Serum neuron-specific enolase as early predictor of outcome after cardiac arrest Crit Care Med 1997 25 1133 1138 10.1097/00003246-199707000-00012 9233737

[B11] Schoerkhuber W Kittler H Sterz F Behringer W Holzer M Frossard M Spitzauer S Laggner AN Time course of serum neuron-specific enolase. A predictor of neurological outcome in patients resuscitated from cardiac arrest Stroke 1999 30 1598 1603 10.1161/01.STR.30.8.1598 10436107

[B12] Rosén H Sunnerhagen KS Herlitz J Blomstrand C Rosengren L Serum levels of the brain-derived proteins S-100 and NSE predict long-term outcome after cardiac arrest Resuscitation 2001 49 183 191 10.1016/S0300-9572(00)00348-8 11382525

[B13] Meynaar IA Oudemans-van Straaten HM van der Wetering J Verlooy P Slaats EH Bosman RJ van der Spoel JI Zandstra DF Serum neuron-specific enolase predicts outcome in post-anoxic coma: a prospective cohort study Intensive Care Med 2003 29 189 195 12594583 10.1007/s00134-002-1573-2

[B14] Tiainen M Roine RO Pettilä V Takkunen O Serum neuron-specific enolase and S-100B protein in cardiac arrest patients treated with hypothermia Stroke 2003 34 2881 2886 10.1161/01.STR.0000103320.90706.35 14631087

[B15] Zingler VC Krumm B Bertsch T Fassbender K Pohlmann-Eden B Early prediction of neurological outcome after cardiopulmonary resuscitation: a multimodal approach combining neurobiochemical and electrophysiological investigations may provide high prognostic certainty in patients after cardiac arrest Eur Neurol 2003 49 79 84 10.1159/000068503 12584414

[B16] Pfeifer R Börner A Krack A Sigusch HH Surber R Figulla HR Outcome after cardiac arrest: predictive values and limitations of the neuroproteins neuron-specific enolase and protein S-100 and the Glasgow Coma Scale Resuscitation 2005 65 49 55 10.1016/j.resuscitation.2004.10.011 15797275

[B17] Rech TH Vieira SR Nagel F Brauner JS Scalco R Serum neuron-specific enolase as early predictor of outcome after in-hospital cardiac arrest: a cohort study Crit Care 2006 10 R133 10.1186/cc5046 16978415 PMC1751053

[B18] Auer J Berent R Weber T Porodko M Lamm G Lassnig E Maurer E Mayr H Punzengruber C Eber B Ability of neuron-specific enolase to predict survival to hospital discharge after successful cardiopulmonary resuscitation CJEM 2006 8 13 18 17175624 10.1017/s1481803500013324

[B19] Reisinger J Höllinger K Lang W Steiner C Winter T Zeindlhofer E Mori M Schiller A Lindorfer A Wiesinger K Siostrzonek P Prediction of neurological outcome after cardiopulmonary resuscitation by serial determination of serum neuron-specific enolase Eur Heart J 2007 28 52 58 17060343 10.1093/eurheartj/ehl316

[B20] Oksanen T Tiainen M Skrifvars MB Varpula T Kuitunen A Castrén M Pettilä V Predictive power of serum NSE and OHCA score regarding 6-month neurologic outcome after out-of-hospital ventricular fibrillation and therapeutic hypothermia Resuscitation 2009 80 165 170 10.1016/j.resuscitation.2008.08.017 18954930

[B21] Martens P Raabe A Johnsson P Serum S-100 and neuron-specific enolase for prediction of regaining consciousness after global cerebral ischemia Stroke 1998 29 2363 2366 10.1161/01.STR.29.11.2363 9804649

[B22] Shinozaki K Oda S Sadahiro T Nakamura M Abe R Nakada TA Nomura F Nakanishi K Kitamura N Hirasawa H! Serum S-100B is superior to neuron-specific enolase as an early prognostic biomarker for neurological outcome following cardiopulmonary resuscitation Resuscitation 2009 80 870 875 10.1016/j.resuscitation.2009.05.005 19535196

[B23] Grubb NR Simpson C Sherwood RA Abraha HD Cobbe SM O'Carroll RE Deary I Fox KA Prediction of cognitive dysfunction after resuscitation from out-of-hospital cardiac arrest using serum neuron-specific enolase and protein S-100 Heart 2007 93 1268 1273 10.1136/hrt.2006.091314 17502328 PMC2000934

[B24] Cummins RO Chamberlain DA Abramson NS Allen M Baskett P Becker L Bossaert L Delooz H Dick W Eisenberg M Recommended guidelines for uniform reporting of data from out-of-hospital cardiac arrest: the Utstein Style. Task Force of the American Heart Association, the European Resuscitation Council, the Heart and Stroke Foundation of Canada, and the Australian Resuscitation Council Ann Emerg Med 1991 20 861 874 1854070

[B25] Le Gall JR Lemeshow S Saulnier F A new Simplified Acute Physiology Score (SAPS II) based on a European/North American multicenter study JAMA 1993 270 2957 2963 10.1001/jama.270.24.2957 8254858

[B26] Knaus WA Draper EA Wagner DP Zimmerman JE Prognosis in acute organ-system failure Ann Surg 1985 202 685 693 10.1097/00000658-198512000-00004 4073980 PMC1250999

[B27] Daubin C Guillotin D Etard O Gaillard C du Cheyron D Ramakers M Bouchet B Parienti JJ Charbonneau P A clinical and EEG scoring system that predicts early cortical response (N20) to somatosensory evoked potentials and outcome after cardiac arrest BMC Cardiovasc Disord 2008 8 35 10.1186/1471-2261-8-35 19055810 PMC2630986

[B28] Synek VM EEG abnormality grades and subdivisions of prognostic importance in traumatic and anoxic coma in adults Clin Electroencephalogr 1988 19 160 166 3416501 10.1177/155005948801900310

[B29] Synek VM Prognostically important EEG coma patterns in diffuse anoxic and traumatic encephalopathies in adults J Clin Neurophysiol 1988 5 161 174 10.1097/00004691-198804000-00003 3074973

[B30] Thömke F Marx JJ Sauer O Hundsberger T Hägele S Wiechelt J Weilemann SL Observations on comatose survivors of cardiopulmonary resuscitation with generalized myoclonus BMC Neurol 2005 5 14 10.1186/1471-2377-5-14 16026615 PMC1190185

[B31] Simel DL Samsa GP Matchar DB Likelihood ratios with confidence: sample size estimation for diagnostic test studies J Clin Epidemiol 1991 44 763 770 10.1016/0895-4356(91)90128-V 1941027

[B32] Levy DE Caronna JJ Singer BH Lapinski RH Frydman H Plum F Predicting outcome from hypoxic-ischemic coma JAMA 1985 253 1420 1426 10.1001/jama.253.10.1420 3968772

[B33] Edgren E Hedstrand U Kelsey S Sutton-Tyrrell K Safar P Assessment of neurological prognosis in comatose survivors of cardiac arrest. BRCT I Study Group Lancet 1994 343 1055 1059 10.1016/S0140-6736(94)90179-1 7909098

[B34] Chen R Bolton CF Young B Prediction of outcome in patients with anoxic coma: a clinical and electrophysiologic study Crit Care Med 1996 24 672 678 10.1097/00003246-199604000-00020 8612421

[B35] Fischer C Luauté J Némoz C Morlet D Kirkorian G Mauguière F Improved prediction of awakening or nonawakening from severe anoxic coma using tree-based classification analysis Crit Care Med 2006 34 1520 1524 10.1097/01.CCM.0000215823.36344.99 16557163

[B36] Jorgensen EO Holm S Resuscitation The natural course of neurological recovery following cardiopulmonary Resuscitation 1998 36 111 122 10.1016/S0300-9572(97)00094-4 9571727

[B37] Madl C Kramer L Domanovits H Woolard RH Gervais H Gendo A Eisenhuber E Grimm G Sterz F Improved outcome prediction in unconscious cardiac arrest survivors with sensory evoked potentials compared with clinical assessment Crit Care Med 2000 28 721 6 10.1097/00003246-200003000-00020 10752821

[B38] Nakabayashi M Kurokawa A Yamamoto Y Immediate prediction of recovery of consciousness after cardiac arrest Intensive Care Med 2001 27 1210 1214 10.1007/s001340100984 11534570

[B39] Robinson LR Micklesen PJ Tirschwell DL Lew HL Predictive value of somatosensory evoked potentials for awakening from coma Crit Care Med 2003 31 960 967 10.1097/01.CCM.0000053643.21751.3B 12627012

[B40] Madl C Grimm G Kramer L Yeganehfar W Sterz F Schneider B Kranz A Schneeweiss B Lenz K Early prediction of individual outcome after cardiopulmonary resuscitation Lancet 1993 341 855 858 10.1016/0140-6736(93)93061-5 8096562

[B41] Rossetti AO Oddo M Logroscino G Kaplan PW Prognostication after cardiac arrest and hypothermia: a prospective study Ann Neurol 2010 67 301 307 20373341 10.1002/ana.21984

[B42] Steffen IG Hasper D Ploner CJ Schefold JC Dietz E Martens F Nee J Krueger A Jörres A Storm C Mild therapeutic hypothermia alters neuron specific enolase as an outcome predictor after resuscitation: 97 prospective hypothermia patients compared to 133 historical non-hypothermia patients Crit Care 2010 14 R69 10.1186/cc8301 20403168 PMC2887191

[B43] Rundgren M Westhall E Cronberg T Rosén I Friberg H Continuous amplitude-integrated electroencephalogram predicts outcome in hypothermia-treated cardiac arrest patients Crit Care Med 2010 38 1838 1844 10.1097/CCM.0b013e3181eaa1e7 20562694

