# Supplementary material for: Serum neuron-specific enolase as predictor of outcome in comatose cardiac-arrest survivors: a prospective cohort study
Source: BMC Cardiovasc Disord. 2011 Aug 8;11:48. doi: 10.1186/1471-2261-11-48 (PMC3161948; doi:10.1186/1471-2261-11-48)

Additional file 1: Receiver operating characteristic curves for 72 h NSE values (ng/mL) to predict poor neurological outcome

Considering 72 h-NSE values (n=61), a level ≥ 68 ng/mL predicted a poor outcome (CPC 4-5) with a positive predictive value of 100% [95% IC = 100% – 100%] and a sensitivity of 67% [95% IC = 54% – 81%].


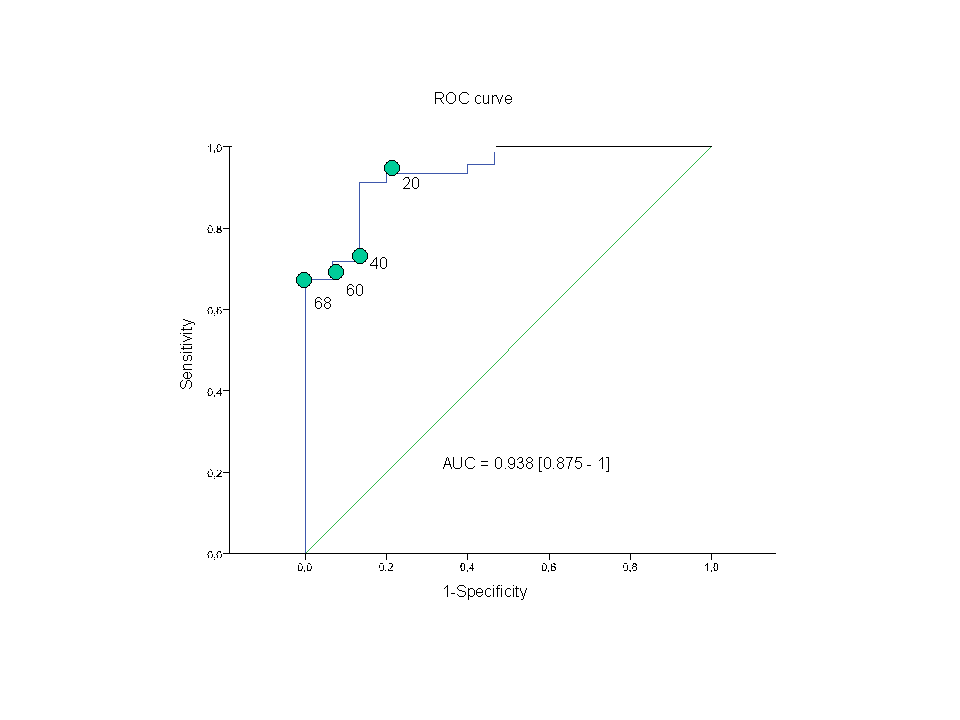

Supplement: Additional file 1 — " Receiver operating characteristic curves for 72 h NSE values (ng/mL) to predict poor neurological outcome. This file highlight that considering 72 h-NSE values (n = 61), a level ≥ 68 ng/mL predicted a poor outcome (CPC 4-5) with a positive predictive value of 100% [95% IC = 100% - 100%] and a sensitivity of 67% [95% IC = 54% - 81%]. [file 1471-2261-11-48-S1.DOC]
